# Aspirin improves transplant-free survival after TIPS implantation in patients with refractory ascites: a retrospective multicentre cohort study

**DOI:** 10.1007/s12072-022-10330-x

**Published:** 2022-04-05

**Authors:** Leon Louis Seifert, Philipp Schindler, Lukas Sturm, Wenyi Gu, Quentin Edward Seifert, Jan Frederic Weller, Christian Jansen, Michael Praktiknjo, Carsten Meyer, Martin Schoster, Christian Wilms, Miriam Maschmeier, Hartmut H. Schmidt, Max Masthoff, Michael Köhler, Michael Schultheiss, Jan Patrick Huber, Dominik Bettinger, Jonel Trebicka, Moritz Wildgruber, Hauke Heinzow

**Affiliations:** 1 Medical Clinic B, Department of Gastroenterology, Hepatology, Endocrinology, Infectiology, University Hospital Muenster, 48149 Muenster, Germany; 2 Clinic for Radiology, University Hospital Muenster, 48149 Muenster, Germany; 3 Department of Medicine II, Medical Center University of Freiburg, University of Freiburg, 79106 Freiburg, Germany; 4 Department of Internal Medicine 1, University Hospital Frankfurt, 60596 Frankfurt, Germany; 5 Georg-August University of Goettingen, 37073 Goettingen, Germany; 6 Department of Hematology, University Hospital Tuebingen, 72076 Tuebingen, Germany; 7 Department of Internal Medicine I, University Hospital Bonn, 53127 Bonn, Germany; 8 Department of Radiology, University Hospital Bonn, 53127 Bonn, Germany; 9 Department of Radiology, University Hospital LMU Munich, 81377 Munich, Germany; 10 Department of Internal Medicine I, Krankenhaus der Barmherzigen Brüder, 54292 Trier, Germany

**Keywords:** Transjugular intrahepatic portosystemic shunt, Decompensated liver cirrhosis, Complications of liver cirrhosis, Portal hypertension, Ascites, Variceal bleeding, Liver transplantation, Hepatic decompensation, Thrombocyte aggregation inhibition, Aspirin, Propensity score matching

## Abstract

**Background and aims:**

Transjugular intrahepatic portosystemic shunt (TIPS) implantation is an established procedure to treat portal hypertension. Impact of administration of aspirin on transplant-free survival after TIPS remains unknown.

**Methods:**

A multicenter retrospective analysis including patients with TIPS implantation between 2011 and 2018 at three tertiary German Liver Centers was performed. *N* = 583 patients were included. Survival analysis was performed in a matched cohort after propensity score matching. Patients were grouped according to whether aspirin was (*PSM-aspirin-cohort*) or was not (*PSM-no-aspirin-cohort*) administered after TIPS. Primary endpoint of the study was transplant-free survival at 12 months after TIPS.

**Results:**

Aspirin improved transplant-free survival 12 months after TIPS with 90.7% transplant-free survival compared to 80.0% (*p* = 0.001) after PSM. Separated by TIPS indication, aspirin did improve transplant-free survival in patients with refractory ascites significantly (89.6% vs. 70.6% transplant-free survival, *p* < 0.001), while no significant effect was observed in patients with refractory variceal bleeding (91.1% vs. 92.2% transplant-free survival, *p* = 0.797).

**Conclusion:**

This retrospective multicenter study provides first data indicating a beneficial effect of aspirin on transplant-free survival after TIPS implantation in patients with refractory ascites.

**Supplementary Information:**

The online version contains supplementary material available at 10.1007/s12072-022-10330-x.

## Introduction

Transjugular intrahepatic portosystemic shunt (TIPS) is performed to reduce portal hypertension and associated complications in patients with decompensated liver cirrhosis [1–3]. The procedure is safe with low rates of complication as a result of major progress in experience and technical ameliorations throughout the last decades [4]. In patients with refractory ascites, TIPS implantation improves transplant-free survival (TFS) and shows superior results of repetitive large volume paracentesis [5–7]. Concerning variceal bleeding, preemptive TIPS implantation should be considered in case of recurrent variceal bleeding as well as acute variceal bleeding [3, 8–10]. Both the European Association for the Study of the Liver (EASL) and the American Association for the Study of Liver Disease (AASLD) recommend TIPS implantation when complications of portal hypertension are present in selected patients [11, 12]. Polytetrafluoroethylene-(PTFE-)-covered stents have improved patency and overall survival compared to the use of bare-metal-stents (BMS) [13]. However, maintaining long-term patency of TIPS remains challenging. Approximately, one-third of patients require invasive TIPS revision to maintain or restore PTFE-shunt-patency within 2 years after placement [13–15]. Shunt stenosis or occlusion mostly occur due to a combination of parenchymal compression, thrombosis formation (acute and chronic) and neointimal hyperplasia [16, 17]. Common guidelines to maintain shunt patency via platelet inhibition or anticoagulative medication are lacking except for patients with portal vein thrombosis or Budd–Chiari syndrome as indication for TIPS [18]. Published experiences and studies are restricted to the era of non-covered stents [19, 20]. Potential beneficial effects of platelet inhibition after in TIPS placement are not sufficiently investigated. With acetylsalicylate acid (aspirin) being established in multiple indications after stent-implantation in the arterial system, little is known about the effect of platelet inhibition in the portal venous system. Administration of aspirin has been shown to be safe in cirrhotic patients [21]. The standardized use of aspirin following TIPS implantation implies potential to reduce TIPS dysfunction and improve post-TIPS survival. Currently, aspirin and other platelet inhibitors are routinely used after TIPS implantation while scientific evidence is lacking [18]. We, therefore, aimed to investigate the effect of aspirin on transplant-free survival (TFS) in patients with TIPS placement in a large retrospective patient cohort.

## Methods

### Study design

Primary endpoint of this retrospective multicenter study was the impact of aspirin on transplant-free survival at 12 months after TIPS implantation.

### Data collection

Patient data from three tertiary care medical centers (University Clinic of Muenster, University Clinic of Bonn, University Medical center of Freiburg, to be called center A, B and C by random assignment) were included. Data were collected retrospectively from all patients in whom TIPS implantation was performed in the institutions between 2011 and 2018. Patient data were collected via electronic record review. Data of a total of 814 patients were available. Laboratory and clinical data before TIPS implantation were assessed within 3 days before TIPS. Follow-up data were collected until death, liver transplantation or end of follow-up.

**Fig. 1:**
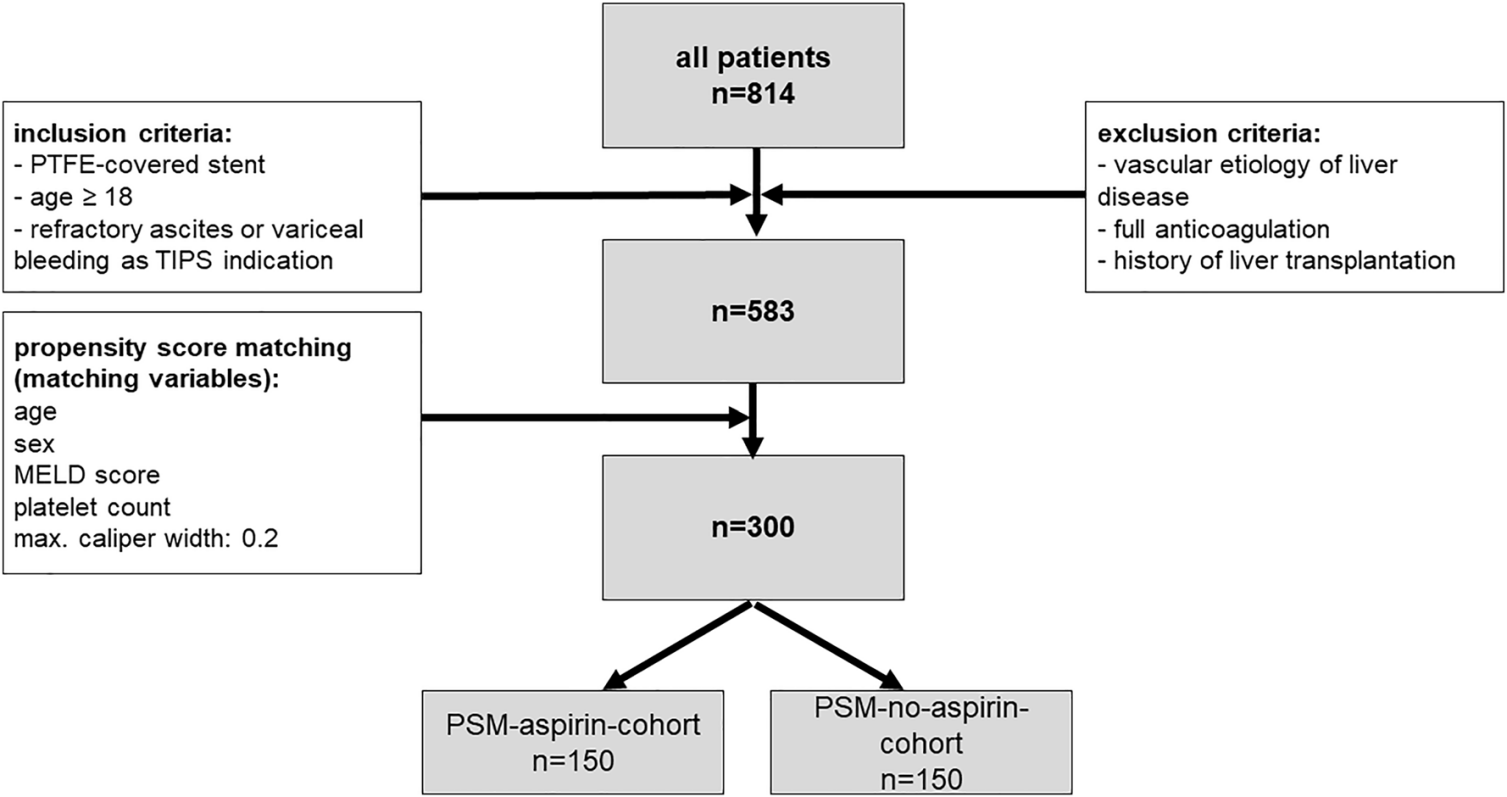
Patient selection and inclusion criteria

For further analysis, inclusion and exclusion criteria were applied (see Fig. 1). All patients receiving TIPS insertion for refractory ascites (defined as ascites refractory to escalated therapy with diuretics and large volume paracentesis) and/or recurrent or refractory esophageal variceal bleeding were included. Patients with other indication for TIPS insertion were excluded. All patients with vascular etiology of liver disease were excluded as well as all patients with full anticoagulation therapy or a history of liver transplantation. Only adult patients (age ≥ 18) in whom PTFE-covered stents (Viatorr. W.L. Gore USA or BeGraft peripheral, Bentley, Hechingen, Germany) were used were included. Transplant-free survival was defined as survival free of death of any cause of liver transplantation. Baseline patient characteristics are presented in Table 1. Administration of aspirin was only performed at institutions A and C as routine care after TIPS implantation if platelet count was > 50 000/µl. Institution B did not administer aspirin following TIPS. Aspirin dosage was 100 mg orally once per day in all patients. Treatment was initiated within 72 h after TIPS implantation irrespective of TIPS indication.

**Table 1:** Baseline characteristics

Parameter	All patients	Aspirin	No-aspirin	*p*-Value
	% (total number) or median/mean (SD)	% (total number) or median/mean (SD)	% (total number) or median/mean (SD)	
n° of patients	583	27.6% (161)	72.4% (422)	–
Center				< 0.001
A	26.4% (153)	34.2% (55)	23.2% (98)	
B	27.2% (159)	–	37.7% (159)	
C	46.4% (271)	65.8% (106)	39.1% (165)	
Sex				0.712
Male	62.8% (366)	64.0% (103)	62.3% (263)	
Female	37.2% (217)	36.0% (58)	37.7% (159)	
Age (median, range, in years)	59 (18–84)	59 (21–81)	59 (18–84)	0.081
PTFE-covered stent	100% (583)	100% (161)	100% (422)	–
Etiology of liver disease				0.292
Alcoholic	58.0% (339)	58.4% (94)	58.1% (245)	
Viral	11.1% (65)	7.5% (12)	12.6% (53)	
NAFLD	8.9% (52)	10.6% (17)	8.3% (35)	
Other	21.9% (127)	23.6% (38)	21.1% (89)	
Child–Pugh grade				0.028
A	21.7% (127)	23.1% (37)	21.1% (90)	
B	59.4% (345)	65.0% (104)	57.2% (241)	
C	18.9% (110)	11.9% (19)	21.6% (91)	
Indication for TIPS				0.121
Ascites	62.3% (364)	65.8% (106)	61.1% (258)	
Variceal bleeding	29.6% (172)	29.8% (48)	29.4% (124)	
Both	8.0% (47)	4.3% (7)	9.5% (40)	
LTX prior TIPS				–
Yes	–	–	–	
No	100% (583)	100% (161)	100% (422)	
HE prior TIPS				0.226
Yes	17.5% (102)	14.3% (23)	18.6% (81)	
No	82.5% (481)	85.7% (138)	81.4% (341)	
Diabetes				0.018
Yes	32.6% (190)	26.0% (58)	31.5% (133)	
No	67.4% (393)	64.0% (103)	68.5% (289)	
Aspirin				< 0.001
Yes	27.6% (161)	100% (161)	–	
No	72.4% (422)		100% (422)	
Anticoagulative regimens				–
Yes	–	–	–	
No	100% (538)	100% (161)	100% (422)	
MELD-score	12.3 (4.9)	11.9 (3.8)	12.6 (5.3)	0.001
MELD-sodium-score	14.0 (5.9)	14.0 (4.9)	14.0 (6.3)	0.119
FIPS	0.08 (1.44)	0.03 (1.62)	0.10 (1.31)	0.001
Bilirubin (mg/dl)	1.40 (1.59)	1.39 (0.96)	1.42 (1.75)	< 0.001
Albumin (g/dl)	3.50 (3.9)	3.7 (3.2)	3.6 (4.2)	< 0.001
Creatinine (mg/dl)	1.06 (0.87)	1.05 (0.63)	1.07 (0.95)	0.182
INR	1.22 (0.23)	1.21 (0.24)	1.24 (0.18)	< 0.001
Platelets (cells/µl)	135 000 (82)	145 000 (90)	133 (75)	< 0.001
Hemoglobin (mg/dl)	10.2 (2.2)	10.4 (2.3)	10.1 (2.2)	0.028
PSG (mmHg)	19.0 (6.0)	19.1 (5.1)	19.0 (6.3)	0.086

### TIPS procedure

TIPS placement was performed by experienced interventional radiologists and/or gastroenterologists in accordance with standard operating procedures at the respective study center. Sonographic guidance was used during the TIPS procedure to control the intrahepatic needle position while gaining access to the portal vein. Portosystemic pressure gradient measurements were done in course of the intervention before and after TIPS implantation to confirm successful reduction of the pressure gradient after TIPS placement. Technical procedures and success rates did not differ between the institutions.

### Statistical analysis

Statistical analyses were performed using *SPSS* version 26.0 (SPSS Inc., Chicago, Illinois, USA) as well as *R* version 3.5.3 (R Foundation for Statistical Computing, Vienna, Austria). All data are presented as the mean (SD), median (range), absolute or percentage, depending on nature of variables and distribution. Chi-square test was used for contingency tables. Paired student t-test was used for quantitative and Mann–Whitney *U* test was used for qualitative data with non-normal distribution. Two-sided *p*-values < 0.05 were defined as statistically significant. For analysis of transplant-free survival after 12 months logistic regression models were created. Variables were consecutively included in a multivariable Cox regression analysis if they were significantly associated with 12-month transplant-free survival in univariate regression model (see Table 2). Multivariable Cox-regression analysis was performed using forward variable selection. For further analysis, we performed propensity score matching (PSM). PSM was performed after logistic regression analysis to create a propensity score for each patient. Age, bilirubin, creatinine, INR and MELD score were identified as suitable variables for PSM (*p* < 0.001). Sex was included to adjust for gender differences. Finally, PSM was performed entering the following variables: age, sex, MELD-score and platelet count. Age, sex and MELD score were included as matching parameters as they included all independent predictors of transplant-free survival identified via logistic and multivariate regression analysis. No significant differences were found if using bilirubin, creatinine and INR or MELD score as matching parameters combined with age and sex. Platelet count was included in further optimization of the matching. Subsequently, a case–control match between patients who received aspirin and patient who did not was obtained by use of nearest-neighborhood-matching using a caliper width of 0.2 without replacement as described elsewhere [22, 23]. A matching ratio of 1:1 was used. Baseline characteristics after PSM are presented in Table 3. Kaplan–Meier curves and the log-rank test were used to analyze the impact on transplant-free survival in the matched cohort.

**Table 2:** Independent predictors of transplant-free survival 12 months after TIPS-placement

Parameter	ß	SE	HR	95% CI for HR	*p*-value
Univariate model					
Age	0.022	0.006	1.022	1.009–1.035	0.001
Indication	0.001	0.108	1.001	0.811–1.236	0.993
PSG before TIPS	–0.002	0.012	0.998	0.974–1.022	0.865
Bilirubin	0.192	0.029	1.211	1.144–1.283	< 0.001
INR	1.263	0.215	3.538	2.322–5.389	< 0.001
Creatinine	0.388	0.057	1.402	1.253–1.569	< 0.001
Albumin	0.004	0.006	1.004	0.992–1.016	0.551
Hemoglobin	–0.094	0.033	0.910	0.854–0.970	0.004
Platelet count	-0.002	0.001	0.998	0.996–1.000	0.035
Diabetes	0.074	0.107	1.076	0.872–1.329	0.494
Aspirin	–0.840	0.280	0.432	0.287–0.648	< 0.001
HE prior TIPS	0.215	0.143	1.240	0.937–1.641	0.132
Etiology of liver disease	–0.106	0.057	0.899	0.804–1.006	0.063
Multivariate model					
Age	0.029	0.007	1.030	1.015–1.044	< 0.001
Aspirin after TIPS	–0.737	0.211	0.479	0.317–0.724	< 0.001
Bilirubin	0.129	0.037	1.138	1.058–1.224	< 0.001
Creatinine	0.268	0.068	1.307	1.144–1.493	< 0.001

**Table 3:** Baseline characteristics grouped by aspirin administration after PSM

Parameter	Aspirin-group	No-aspirin-group	Cohen’s *d*	*p*-value
	% (total number) or median/mean (SD)	% (total number) or median/mean (SD)		
n° of patients	50% (150)	50% (150)		–
Center			–	< 0.001
A	34.0% (51)	21.3% (32)		
B	–	39.3% (59)		
C	66.0% (99)	39.3% (59)		
Sex			0.073	0.633
Male	64.0% (96)	61.3% (92)		
Female	34.0% (54)	38.7% (58)		
Age (median, range, in years)	60 (21–81)	60 (26–82)	− 0.021	0.811
PTFE-covered stent	100% (150)	100% (150)	–	–
Etiology of liver disease			0.109	0.941
Alcoholic	58.0% (87)	57.3% (86)		
Viral	8.0% (12)	10.0% (15)		
NAFLD	10.9% (15)	10.0% (15)		
Other	24.0% (36)	22.7% (34)		
Child–Pugh grade			0.029	0.246
A	24.0% (36)	28.2% (42)		
B	64.0% (96)	61.7% (92)		
C	12.0% (18)	10.1% (18)		
Indication for TIPS			0.043	0.371
Ascites	65.3% (98)	62.0% (93)		
Variceal bleeding	30.7% (46)	29.3% (44)		
Both	4.0% (6)	8.7% (13)		
LTX prior TIPS			0.077	0.665
Yes	–	–		
No	100% (150)	100% (150)		
HE prior TIPS			0.080	0.690
Yes	13.3% (20)	14.0% (21)		
No	86.7% (130)	86.0% (129)		
Diabetes			0.031	0.267
Yes	36.7% (55)	34.0% (54)		
No	63.3% (95)	64.0% (96)		
Platelet inhibitors			–	< 0.001
Yes	100% (150)	–		
No	–	100% (150)		
Anticoagulative regimens			-	-
Yes	–	–		
No	100% (150)	100% (150)		
MELD-score	11.7 (3.5)	11.7 (3.6)	< 0.001	0.819
MELD-sodium-score	14.1 (4.7)	13.4 (5.4)	0.138	0.244
FIPS	− 0.22 (0.86)	− 0.23 (0.96)	− 0.011	0.883
Bilirubin (mg/dl)	1.36 (0.96)	1.42 (0.86)	− 0.066	0.418
Albumin (g/dl)	3.6 (3.2)	3.7 (3.3)	− 0.031	0.670
Creatinine (mg/dl)	1.04 (0.58)	1.02 (0.61)	0.034	0.734
INR	1.21 (0.18)	1.21 (0.17)	< 0.001	0.695
Platelets (cells/µl)	159 000 (82)	159 000 (67)	< 0.001	0.756
Hemoglobin (mg/dl)	11.2 (3.6)	10.8 (2.3)	0.13	0.336
PSG (mmHg)	19.2 (5.1)	20.0 (5.9)	− 0.14	0.130

## Results

All available patient data from patients receiving TIPS implantation from the three participating institutions were collected (*n* = 814 patients). After application of exclusion and inclusion criteria, data of a total of 583 patients were included in the final analysis as presented in Fig. 1. Baseline characteristics of the entire patient cohort are presented in Table 1 and separated by institution in supplementary Table 1.

To identify independent risk factors associated with impaired transplant-free survival after TIPS implantation, we performed multivariate Cox regression analysis using forward variable selection with all variables that were significantly associated with 12-month transplant-free survival in univariable regression analysis. Concerning laboratory parameters before TIPS placement, we identified increased levels of bilirubin (*p* < 0.001), creatinine (*p* < 0.001) and higher age (*p* < 0.001) as risk factors for death or liver transplantation after TIPS, whereas administration of aspirin (*p* < 0.001) is an independent predictor of transplant-free survival at 12 months (see Table 2).

Due to significant differences between patients who received aspirin and did not (see Table 1), comparison of transplant-free survival in a matched patient-cohort was necessary to investigate the beneficial effect of aspirin on transplant-free survival. We performed propensity score matching analysis using age, sex, MELD-score and platelet count as matching parameters. The baseline characteristics of the matched patient cohort are presented in Table 3. Patients were grouped based on aspirin administration (*PSM-aspirin-cohort, PSM-no-aspirin-cohort*). There were no significant differences concerning etiology or severity of liver disease in the matched patient cohort. Satisfactory balance of respective variables is indicated by Cohen’s d.

**Fig. 2:**
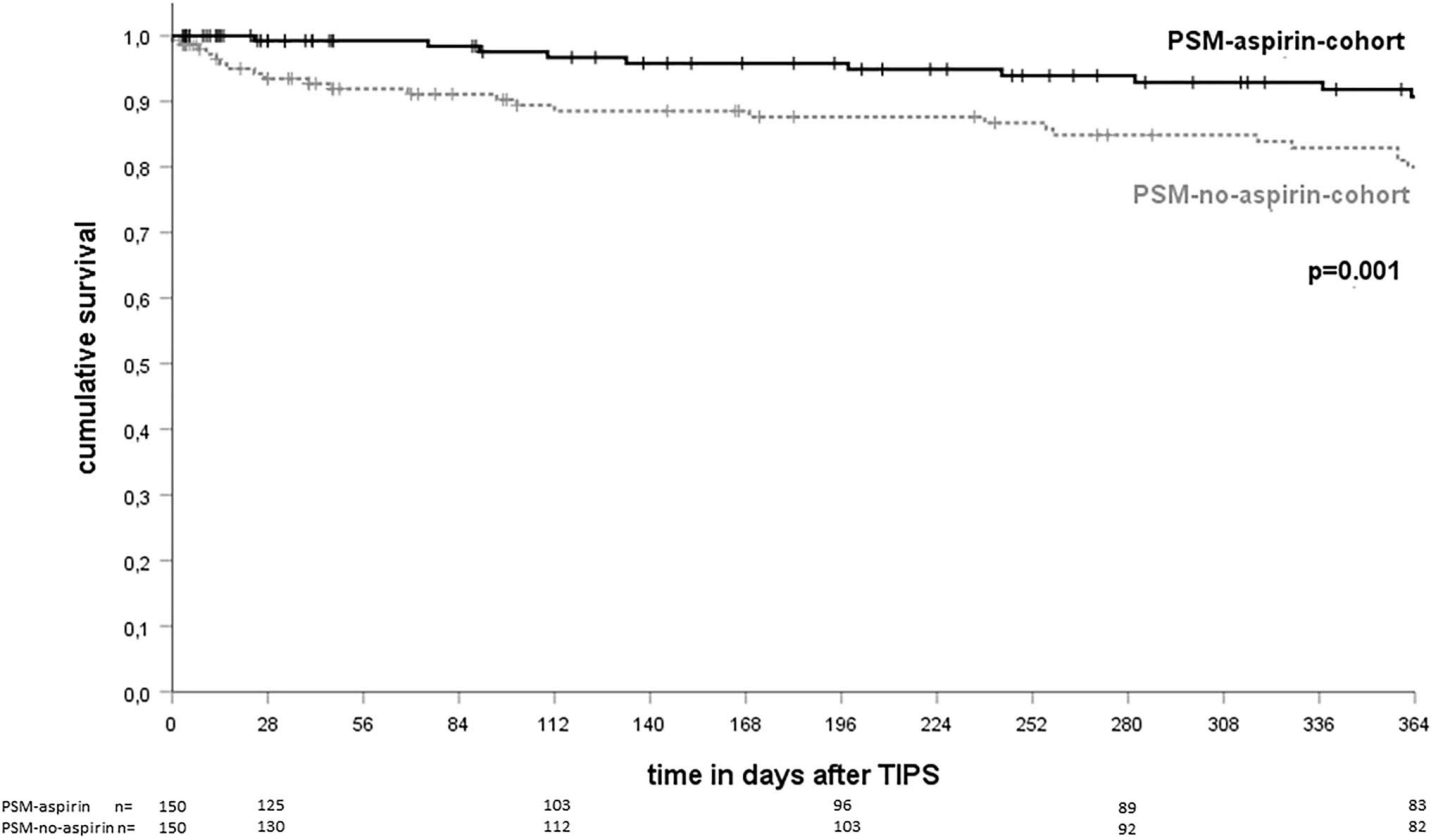
Transplant-free survival after TIPS implantation after PSM. A Transplant-free survival 12 months after TIPS-placement was 90.7% in the *PSM-aspirin-cohort* and 80.0% in the *PSM-no-aspirin-cohort* (Kaplan–Meier curve, *p* = 0.001, log-rank test). + , censored patients

Kaplan–Meier analysis shows superior transplant-free survival in patients who received aspirin after TIPS implantation (*p* = 0.001, log-rank test; see Fig. 2). In the *PSM-aspirin-cohort*, 97.6%, 95.8% and 90.7% patients achieved transplant-free survival at 3, 6 and 12 months after TIPS implantation, respectively, compared to 90.2%, 87.6% and 80.0% in the *PSM-no-aspirin-cohort*. Transplant-free survival did not differ significantly between the different centers included irrespective of aspirin administration (*p* = 0.424 and *p* = 0.272 respectively, log-rank test). Improvement of transplant-free survival by aspirin was pronounced in more severe cirrhosis (Child B and C cirrhosis, *p* = 0.007, log-rank test; see supplementary Fig. 1B) compared to patients with Child A cirrhosis (*p* = 0.064, log-rank test; see supplementary Fig. 1A).

**Fig. 3:**
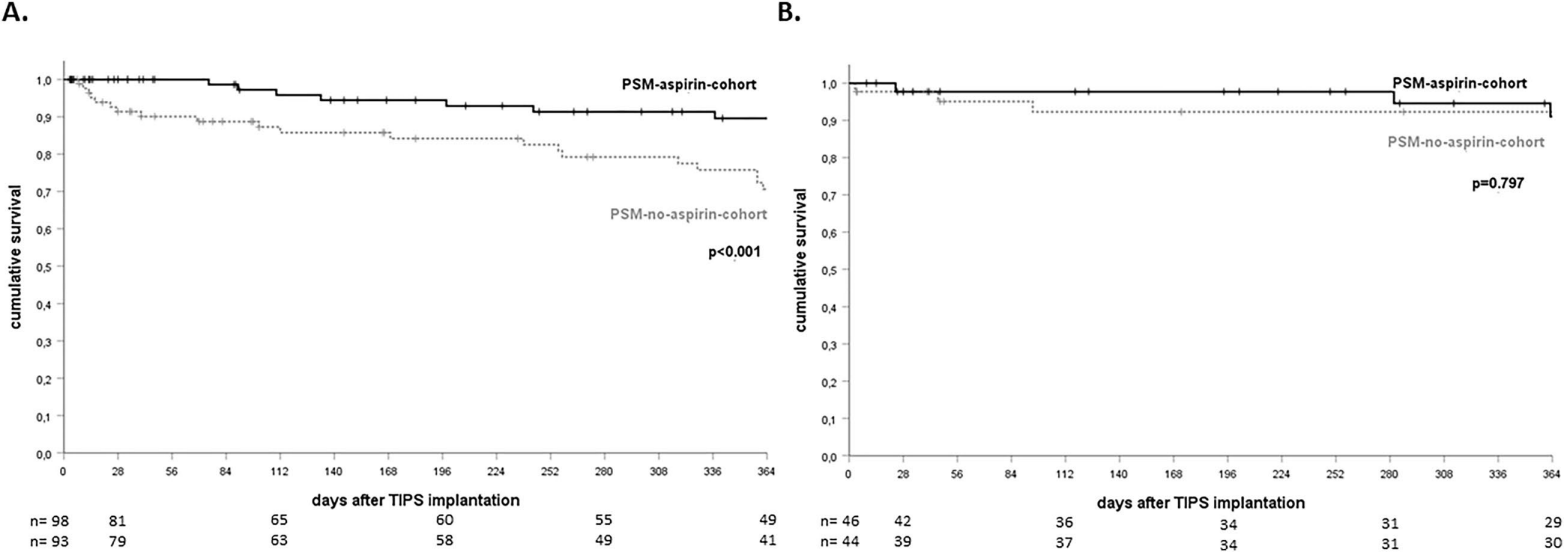
Transplant-free survival by TIPS indication after PSM. **a** Transplant-free survival 12 months after TIPS-placement among patients with refractory ascites as TIPS indication was 89.6% in the *PSM-aspirin-cohort* and 70.6% in the *PSM-no-aspirin-cohort* (Kaplan–Meier curve, *p* < 0.001, log-rank test). **b** Transplant-free survival 12 months after TIPS-placement among patients with variceal bleeding as TIPS indication was 91.1% in the *PSM-aspirin-cohort* and 92.2% in the *PSM-no-aspirin-cohort* (Kaplan–Meier curve, *p* = 0.797, log-rank test). + , censored patients

Survival rates are distinct by TIPS indication. Baseline characteristics according to TIPS indication in the matched cohort are presented in supplementary tables 2 and 3. In patients with refractory ascites as indication for TIPS implantation (total n = 191 patients; 98 *PSM-aspirin-cohort*, 93 *PSM-no-aspirin cohort*), administration of aspirin shows significant improvement of transplant-free survival after 12 months (89.6% in the *PSM-aspirin-cohort*, 70.6% in the *PSM-no-aspirin-cohort*, *p* < 0.001, log-rank test; see Fig. 3a). On the other hand, transplant-free survival was not significantly affected in patients with variceal bleeding as TIPS indication (total *n* = 90 patients; 46 *PSM-aspirin-cohort*, 44 *PSM-no-aspirin cohort*) with a survival rate of 91.1% in the *PSM-*aspirin-cohort compared to 92.2% in the *PSM-no-aspirin-cohort* (*p* = 0.797, log-rank test; see Fig. 3b). No significant effect of aspirin was observed in patients in whom TIPS indication was not clearly distinguishable between refractory ascites and refractory variceal bleeding (total *n* = 19 patients; 6 *PSM-aspirin-cohort*, 13 *PSM-no-aspirin cohort)* (*p* = 0.297, log-rank test, data not visualized).

## Discussion

This multicenter retrospective study found a beneficial effect of aspirin on transplant-free survival in patients who received TIPS implantation in a real-life cohort including 583 patients from three major German tertiary care liver centers. Aspirin was associated with a significant superior transplant-free survival within the first 12 months after TIPS implantation. We confirmed these findings through a robust matching using propensity score matching method. No significant differences concerning established parameters of liver function (Child–Pugh Score, MELD-score) or recently introduced parameters of survival after TIPS implantation (FIPS-score) was found between the created cohorts [24]. The beneficial effect of aspirin is dependent from the underlying TIPS indication since transplant-free survival was improved in patients with refractory ascites but not in patients with variceal bleeding.

To the best of our knowledge, there are no published studies that investigate the effect of aspirin on transplant-free survival in the era of PTFE-covered stents. The question of whether to administer anticoagulation medication or platelet inhibitors to prevent TIPS associated complications remains unanswered and respective strategies differ immensely [18]. The effects of prophylactic anticoagulation by administration of low molecular weight heparin (enoxaparin or nadroparin) after TIPS-implantation are currently under investigation in a prospective study [25]. Current published evidence in this field is sparse and not sufficient to develop reliable recommendations.

In stent placement in arterial systems, administration of platelet activation inhibitors is established. In patients with TIPS implantation platelet activation inhibition appears to be a promising target, too. Altered platelet activation has been shown to be present in patients with liver cirrhosis. A platelet activating state can precisely be described in the portal venous system of cirrhotic patients. Portal hypertension facilitates bacterial translocation and increases oxidative stress. Subsequently, several increased markers of platelet activation create a possibly prothrombotic environment as shown in the portal venous blood of patients undergoing TIPS implantation [26].

The effect of aspirin on patients after TIPS implantation has been studied before in a small prospective study (*n* = 44) in the era of bare-metal stents. At that time, no significant difference was found concerning shunt patency 3 months after TIPS placement. Importantly, 3-month administration of aspirin did not increase risk of rebleeding in this cohort consisting of almost 90% of patients receiving TIPS for recurrent variceal bleeding [27]. The same group later found beneficial effects of phenprocoumon (target INR 1.7–2.1) on shunt patency [20]. Periprocedural application of heparin was also shown to prevent shunt insufficiency [28]. PTFE-covering later improved prevention of development of pseudo-intimal hyperplasia and stent stenosis resulting in a much higher primary patency rate [13]. The discussed studies were performed before introduction of PTFE-stents and the results are not applicable on today’s patients.

Interestingly, transplant-free survival was only significantly improved in patients with refractory ascites as TIPS indication and not affected in patients with refractory variceal bleeding. It is known that patient with refractory ascites represent a cohort of more advanced cirrhosis [29]. Consequently, transplant-free survival after TIPS is also impaired in these patients compared to patients with variceal bleeding as indication for TIPS insertion and even differential cutoff values in prognostic-tools have been proposed [24]. In our study too, patients with variceal bleeding as TIPS indication show less advanced cirrhosis (see supplementary tables 2 and 3). Regardless of TIPS indication, aspirin did not improve survival significantly in patients with Child A cirrhosis. In addition, aspirin potentially increased the rate of rebleeding in those patients. Our study does not include data on adverse events of aspirin after TIPS placement to further investigate these hypotheses to explain the differential effect of aspirin after TIPS insertion. The lack of information concerning treatment adherence represents a further potential bias. The effects of other drugs with a positive influence on patient survival in cirrhotic patients (lactulose, statins, antibiotics etc.) have not been studied in this analysis as the respective data are unavailable. It is furthermore important to outline that the observed improvement of survival may not exclusively be due to effects of aspirin on the hepatic system. The higher number of censors in the *PSM-aspirin-cohort* possibly affects the results. Despite a statistically robust PSM-matching creating comparable patient cohorts according to aspirin use with no significant differences in baseline characteristics, an influence of the significant differences in the unmatched cohorts cannot be ruled out. In addition to possible imbalance of unknown and unmeasured confounders, this represents a major bias to our study in comparison to prospective randomized controlled trials [30].

Interestingly, the protective effect of aspirin on transplant-free survival occurs early after TIPS procedure. This may be explainable due the mortality being highest within the first 100 days after TIPS insertion specifically in patients with refractory ascites [31]. Causes of death were not analyzed separately in the presented study due to unavailability of data. However, application of aspirin implicates further potential beneficial effects in patients in liver cirrhosis after TIPS placement. In a cross-sectional analysis in patients with chronic liver disease, use of aspirin was associated with a lower index liver fibrosis [32]. In a prospective study, a beneficial effect of aspirin was confirmed as aspirin was associated with less severe liver injury in NAFLD and NASH and decreased risk of fibrosis progression [33]. In a nationwide study including all Swedish patients with viral hepatitis due to hepatitis B or hepatitis C infection, aspirin administration was even associated with a decreased liver-related mortality and decreased incidence of hepatocellular carcinoma without increasing the probability of gastrointestinal bleeding [34]. Clearly, these findings are individually insufficient to explain a superior survival already at 12 months after TIPS implantation as seen in our study. Information on TIPS shunt patency rates or recurrence rates of initial TIPS indications is lacking in our cohort. Thus, a beneficial effect of aspirin on the general disease progression in cirrhotic patients beyond direct effect concerning TIPS patency cannot be excluded. It is assumable that the beneficial effect of aspirin in TIPS patients results from a cumulation of the described effects on platelet activation, reduced progression of liver fibrosis and anticancerogenic effects. A longer follow-up period is needed to confirm these findings and a prospective study with detailed analysis of causes of death and adverse events is desirable.

The retrospective design of this study limits the reliability of its results. Due to the retrospective character of this study, patient-based differences in the decision whether aspirin was administered or not cannot be excluded. Despite well-balanced propensity score matching, confounding in treatment allocation may be underestimated in the matched cohorts. A selection bias of patients cannot be excluded. Heterogeneity of the respective patient cohort and medical regimen at the different institutions possibly affect the results.

In conclusion, this retrospective multicenter cohort study provides first evidence that aspirin administration after TIPS implantation has a substantial effect on transplant-free survival in patients with refractory ascites as TIPS indication. Our findings support the necessity for prospective randomized clinical trials to investigate the effects of aspirin in TIPS patients.

## Supplementary Information

Below is the link to the electronic supplementary material.

Supplementary figure 1: Transplant-free survival by severity of liver cirrhosis A. Transplant-free survival 12 months after TIPS-placement among patients with Child A cirrhosis was 100% in the PSM-aspirin-cohort and 86.4% in the PSM-no-aspirin-cohort (Kaplan-Meier curve, p=0.064, log-rank test). B. Transplant-free survival 12 months after TIPS-placement among patients with Child B or C cirrhosis was 87.5% in the PSM-aspirin-cohort and 78.2% in the PSM-no-aspirin-cohort (Kaplan-Meier curve, p=0.007, log-rank test). (TIF 90 kb)

Supplementary file2 (DOCX 18 kb)

Supplementary file3 (DOCX 17 kb)

Supplementary file4 (DOCX 17 kb)

## Abbreviations

ß: Regression coefficient
FIPS: Freiburg-index of post-TIPS survival
HCC: Hepatocellular carcinoma
HE: Hepatic encephalopathy
HR: Hazard ratio
INR: International normalized ratio
LTX: Liver transplantation
MELD: Model for end-stage liver disease
m: Matched
NAFLD: Non-alcoholic fatty liver disease
NASH: Non-alcoholic steatohepatitis
PSG: Portosystemic pressure gradient
PSM: Propensity score matching
PTFE: Polytetrafluoroethylene
SD: Standard deviation
SE: Standard error
TFS: Transplant-free survival
TIPS: Transjugular intrahepatic portosystemic shunt
95% CI: 95% Confidence interval
