# Supplementary figures and images for: Aspirin improves transplant-free survival after TIPS implantation in patients with refractory ascites: a retrospective multicentre cohort study
Source: Hepatol Int. 2022 Apr 5;16(3):658–68. doi: 10.1007/s12072-022-10330-x (PMC9174324; doi:10.1007/s12072-022-10330-x)

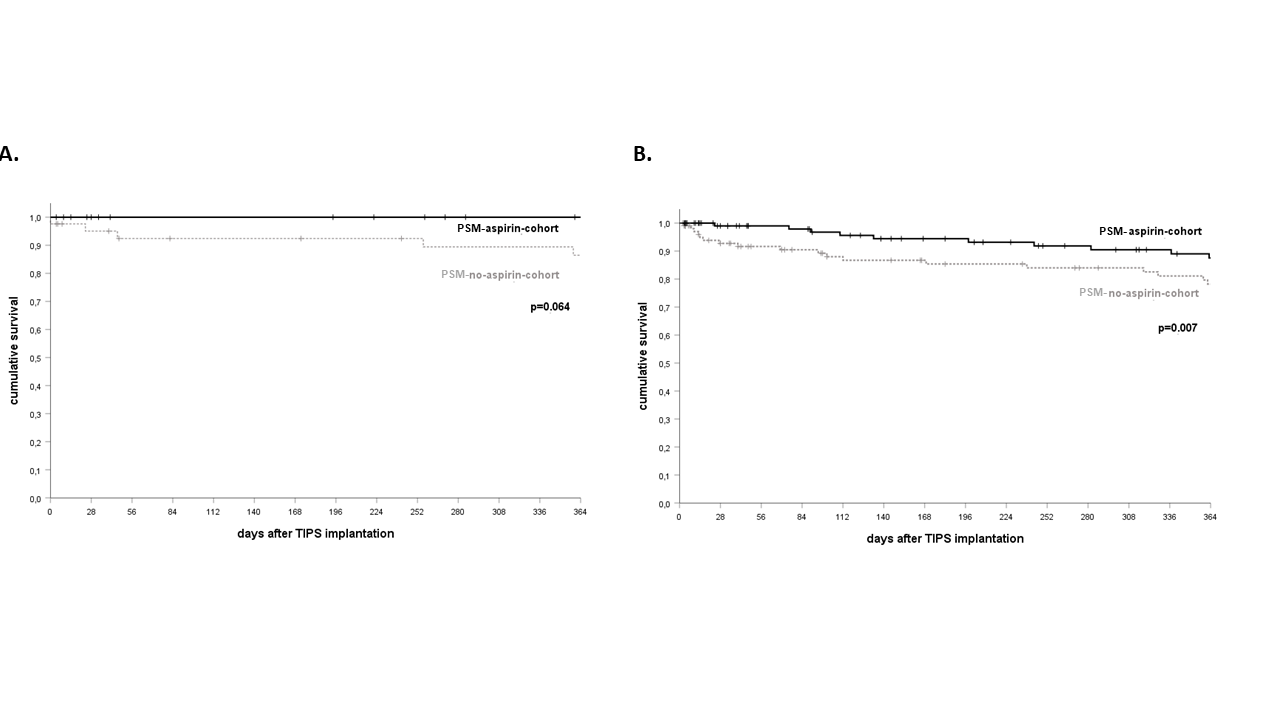

Supplement: Supplementary file 1 — Supplementary figure 1: Transplant-free survival by severity of liver cirrhosis A. Transplant-free survival 12 months after TIPS-placement among patients with Child A cirrhosis was 100% in the PSM-aspirin-cohort and 86.4% in the PSM-no-aspirin-cohort (Kaplan-Meier curve, p=0.064, log-rank test). B. Transplant-free survival 12 months after TIPS-placement among patients with Child B or C cirrhosis was 87.5% in the PSM-aspirin-cohort and 78.2% in the PSM-no-aspirin-cohort (Kaplan-Meier curve, p=0.007, log-rank test). (TIF 90 kb) [file 12072_2022_10330_MOESM1_ESM.tif]
