# Supplementary material for: Aspirin improves transplant-free survival after TIPS implantation in patients with refractory ascites: a retrospective multicentre cohort study
Source: Hepatol Int. 2022 Apr 5;16(3):658–68. doi: 10.1007/s12072-022-10330-x (PMC9174324; doi:10.1007/s12072-022-10330-x)
Supplement: Supplementary file 2 — Supplementary file2 (DOCX 18 kb) [file 12072_2022_10330_MOESM2_ESM.docx]

**Supplementary table 1:**

| parameter | Center 1  % (total number) or median/mean (SD) | Center 2  % (total number) or median/  mean (SD) | Center 3  % (total  number)  or median/  mean (SD) | p-value |
| --- | --- | --- | --- | --- |
| n° of patients | 154 | 159 | 271 | - |
| center  A  B  C | 100% (154)  -  - | -  100% (159)  - | -  -  100% (271) | - |
| sex  male  female | 61.7% (59)  38.3% (59) | 59.1% (94)  40.9% (65) | 65.7% (178)  34.3% (93) | 0.374 |
| age (median, range, in y) | 58 (18-84) | 59 (21-81) | 59 (18-84) | 0.050 |
| PTFE-covered stent | 100% (154) | 100% (159) | 100% (271) | - |
| etiology of liver disease  alcoholic  viral  NAFLD  other | 51.9% (90)  8.4% (13)  11.7% (18)  27.9% (143) | 60.4% (96)  8.8% (14)  3.8% (6)  27.0% (43) | 60.1% (163)  14.0% (38)  10.3% (28)  15.5% (42) | 0.002 |
| Child-Pugh grade  A  B  C | 26.0% (40)  61.7% (95)  12.3% (19) | 23.1% (28)  65.6% (104)  16.8% (27) | 21.2% 56)  54.9% (145)  23.9% (63) | 0.002 |
| indication for TIPS  ascites  variceal bleeding  both | 50.0% (77)  33.1% (51)  16.9% (26) | 62.9% (100)  26.4% (42)  10.7% (17) | 89.0% (187)  29.5% (80)  1.5% (4) | <0.001 |
| LTX prior TIPS  yes  no | -  100% (154) | -  100% (159) | -  100% (271) | - |
| HE prior TIPS  yes  no | 24.7% (38)  75.3% (116) | 13.9% (23)  86.1% (136) | 15.5% (41)  84.5% (230) | 0.014 |
| diabetes  yes  no | 32.5% (50)  67.5% (104) | 31.0% (49)  69.0% (110) | 33.6% (91)  66.4% (180) | 0.018 |
| aspirin  yes  no | 36.7% (55)  63.3% (95) | 100% (159)  - | 41% (111)  59% (160) | <0.001 |
| anticoagulative regimens  yes  no | -  100% (154) | -  100% (159) | -  100% (422) | - |
| MELD-score | 12.4 (5.5) | 12.3 (5.1) | 12.8 (4.5) | <0.001 |
| MELD-sodium-score | 14.0 (6.2) | 13.9 (6.2) | 15.0 (5.6) | 0.119 |
| FIPS | 0.09 (1.23) | 0.20 (1.55) | 0.02 (1.46) | <0.001 |
| bilirubin (mg/dl) | 1.30 (2.22) | 1.52 (1.46) | 1.21 (1.17) | 0.001 |
| albumin (g/dl) | 3.3 (3.3) | 3.1 (3.2) | 3.4 (2.5) | <0.001 |
| creatinine (mg/dl) | 1.10 (0.87) | 0.96 (0.82) | 1.07 (0.90) | 0.182 |
| INR | 1.30 (0.23) | 1.27 (0.23) | 1.22 (0.19) | <0.001 |
| platelets (ths/µl) | 129 (77) | 141 (87) | 137 (80) | <0.001 |
| hemoglobin (mg/dl) | 10.4 (2.1) | 9.8 (2.2) | 10.3 (2.3) | 0.028 |
| PSG (mmHg) | 18.8 (5.8) | 20.2 (6.1) | 20.3 (5.5) | 0.086 |

**Supplementary table 1: Baseline characteristics by affiliation**
