# Supplementary material for: Aspirin improves transplant-free survival after TIPS implantation in patients with refractory ascites: a retrospective multicentre cohort study
Source: Hepatol Int. 2022 Apr 5;16(3):658–68. doi: 10.1007/s12072-022-10330-x (PMC9174324; doi:10.1007/s12072-022-10330-x)
Supplement: Supplementary file 3 — Supplementary file3 (DOCX 17 kb) [file 12072_2022_10330_MOESM3_ESM.docx]

**Supplementary table 2:**

| parameter | aspirin-group  % (total number) or median/mean (SD) | no-aspirin-group  % (total number) or median/mean (SD) | p-value |  |
| --- | --- | --- | --- | --- |
| n° of patients | 50% (98) | 50% (93) | - | |
| center  A  B  C | 28.6% (28)  -  71.4% (70) | 14.0% (13)  44.1% (41)  41.9% (39) | <0.001 | |
| sex  male  female | 64.3% (63)  35.7% (35) | 66.7% (62)  33.3% (31) | 0.729 | |
| age (median, range, in y) | 62 (21-81) | 60 (26-82) | 0.792 | |
| PTFE-covered stent | 100% (98) | 100% (93) | - | |
| etiology of liver disease  alcoholic  viral  NAFLD  other | 64.3% (63)  8.2% (8)  11.2% (11)  16.3% (16) | 67.7% (63)  7.5% (7)  8.6% (8)  16.1% (15) | 0.442 | |
| Child-Pugh grade  A  B  C | 16.3% (16)  70.4% (69)  13.3% (13) | 17.4% (16)  73.9% (68)  8.7% (8) | 0.077 | |
| indication for TIPS  ascites  variceal bleeding  both | 100% (98)  -  - | 100% (93)  -  - | - | |
| LTX prior TIPS  yes  no | -  100% (98) | -  100% (93) | - | |
| HE prior TIPS  yes  no | 16.3% (16)  83.7% (82) | 11.8% (11)  88.2% (82) | 0.552 | |
| diabetes  yes  no | 38.8% (38)  61.2% (60) | 38.7% (36)  61.3% (57) | 0.339 | |
| aspirin  yes  no | 100% (98)  - | -  100% (93) | <0.001 | |
| anticoagulative regimen  yes  no | -  100% (98) | -  100% (93) | - | |
| MELD-score | 12.4 (0.35) | 12.2 (3.82) | 0.307 | |
| MELD-sodium-score | 15.4 (0.39) | 15.1 (5.43) | 0.625 | |
| FIPS | 0.08 (0.74) | 0.10 (0.82) | 0.308 | |
| bilirubin (mg/dl) | 1.47 (1.00) | 1.40 (0.80) | 0.626 | |
| Albumin (g/dl) | 3.2 (2.9) | 3.2 (2.7) | 0.670 | |
| creatinine (mg/dl) | 1.38 (0.64) | 1.34 (0.17) | 0.734 | |
| INR | 1.18 (0.17) | 1.22 (0.67) | 0.114 | |
| platelets (ths/µl) | 166 (67) | 154 (96) | 0.074 | |
| Hemolobin (mg/dl) | 10.5 (2.3) | 10.3 (1.99) | 0.640 | |
| PSG (mmHg) | 19.8 (4.8) | 20.8 (6.1) | 0.130 | |

**Supplementary table 2: Baseline characteristics of patients with refractory ascites as TIPS indication in the matched cohort**

Abbreviations: PTFE, polytetrafluoroethylene; NAFLD: non-alcoholic fatty liver disease; MELD, model of endstage liver disease; INR, international normalized ratio; FIPS, Freiburg-Index of post-TIPS survival; PSG, portosystemic pressure gradient; TIPS, transjugular intrahepatic portosystemic shunt; LTX, liver transplantation; HE, hepatic encephalopathy; aspirin, acetylsalicylate acid;
