# Supplementary material for: Aspirin improves transplant-free survival after TIPS implantation in patients with refractory ascites: a retrospective multicentre cohort study
Source: Hepatol Int. 2022 Apr 5;16(3):658–68. doi: 10.1007/s12072-022-10330-x (PMC9174324; doi:10.1007/s12072-022-10330-x)
Supplement: Supplementary file 4 — Supplementary file4 (DOCX 17 kb) [file 12072_2022_10330_MOESM4_ESM.docx]

**Supplementary table 3**

| parameter | aspirin-group  % (total number) or median/mean (SD) | no-aspirin-group  % (total number) or median/mean (SD) | p-value |  |
| --- | --- | --- | --- | --- |
| n° of patients | 50% (46) | 50% (44) | - | |
| center  A  B  C | 41.3% (19)  -  58.7% (27) | 25.0% (11)  34.1% (15)  40.9% (18) | <0.001 | |
| sex  male  female | 60.9% (28)  39.1% (18) | 52.3% (23)  47.7% (21) | 0.211 | |
| age (median, range, in y) | 62 (21-81) | 60 (26-82) | 0.832 | |
| PTFE-covered stent | 100% (46) | 100% (44) | - | |
| etiology of liver disease  alcoholic  viral  NAFLD  other | 47.8% (22)  6.5% (3)  6.5% (3)  39.5% (18) | 36.4% (16)  13.6% (6)  13.6% (6)  36.4% (16) | 0.388 | |
| Child-Pugh grade  A  B  C | 65.2% (30)  28.3% (13)  6.5% (3) | 50.0% (22)  43.2% (19)  6.8% (3) | 0.315 | |
| indication for TIPS  ascites  variceal bleeding  both | -  100% (46)  - | -  100% (44)  - | - | |
| LTX prior TIPS  yes  no | -  100% (46) | -  100% (44) | - | |
| HE prior TIPS  yes  no | 8.7% (4)  91.3% (42) | 15.9% (7)  84.1% (37) | 0.296 | |
| diabetes  yes  no | 34.8% (16)  65.2% (30) | 34.1% (15)  65.9% (29) | 0.775 | |
| aspirin  yes  no | 100% (46)  - | -  100% (44) | <0.001 | |
| anticoagulative regimen  yes  no | -  100% (46) | -  100% (44) | - | |
| MELD-score | 9.9 (3.56) | 10.0 (2.69) | 0.263 | |
| MELD-sodium-score | 11.4 (4.8) | 10.7 (3.6) | 0.064 | |
| FIPS | -0.69 (0.91) | -0.71 (0.82) | 0.915 | |
| bilirubin (mg/dl) | 1.01 (0.94) | 1.05 (0.94) | 0.598 | |
| Albumin (g/dl) | 3.6 (3.9) | 3.7 (3.8) | 0.190 | |
| creatinine (mg/dl) | 0.90 (0.32) | 0.89 (0.34) | 0.233 | |
| INR | 1.19 (0.14) | 1.18 (0.21) | 0.644 | |
| platelets (ths/µl) | 147 (61) | 139 (45) | 0.059 | |
| Hemolobin (mg/dl) | 10.0 (2.3) | 10.1 (2.5) | 0.283 | |
| PSG (mmHg) | 19.1 (5.8) | 18.9 (5.8) | 0.856 | |

**Supplementary table 3: Baseline characteristics of patients with refractory variceal bleeding as TIPS indication in the matched cohort**

Abbreviations: PTFE, polytetrafluoroethylene; NAFLD: non-alcoholic fatty liver disease; MELD, model of endstage liver disease; INR, international normalized ratio; FIPS, Freiburg-Index of post-TIPS survival; PSG, portosystemic pressure gradient; TIPS, transjugular intrahepatic portosystemic shunt; LTX, liver transplantation; HE, hepatic encephalopathy; aspirin, acetylsalicylate acid;
